# A comparison of hydration effect on body fluid and temperature regulation between Malaysian and Japanese males exercising at mild dehydration in humid heat

**DOI:** 10.1186/1880-6805-33-5

**Published:** 2014-02-04

**Authors:** Hitoshi Wakabayashi, Titis Wijayanto, Joo-Young Lee, Nobuko Hashiguchi, Mohamed Saat, Yutaka Tochihara

**Affiliations:** 1 Department of Human Science, Kyushu University, Fukuoka, Japan; 2 Faculty of Engineering, Chiba Institute of Technology, 2-1-1, Shibazono, Narashino, Chiba 275-0023, Japan; 3 Department of Clothing and Textiles, Seoul National University, Seoul, Korea; 4 Department of Health Science, Faculty of Medicine, Kyushu University, Fukuoka, Japan; 5 School of Health Sciences, Universiti Sains Malaysia, Kelantan, Malaysia; 6 The Open University of Japan, Fukuoka, Japan

**Keywords:** Body fluid regulation, Heat acclimatization, Hypohydration, Plasma volume, Thermoregulation

## Abstract

**Background:**

This study investigated the effect of hydration differences on body fluid and temperature regulation between tropical and temperate indigenes exercising in the heat.

**Methods:**

Ten Japanese and ten Malaysian males with matched physical characteristics (height, body weight, and peak oxygen consumption) participated in this study. Participants performed exercise for 60 min at 55% peak oxygen uptake followed by a 30-min recovery at 32°C and 70% relative air humidity with hydration (4 times each, 3 mL per kg body weight, 37°C) or without hydration. Rectal temperature, skin temperature, heart rate, skin blood flow, and blood pressure were measured continuously. The percentage of body weight loss and total sweat loss were calculated from body weight measurements. The percentage change in plasma volume was estimated from hemoglobin concentration and hematocrit.

**Results:**

Malaysian participants had a significantly lower rectal temperature, a smaller reduction in plasma volume, and a lower heart rate in the hydrated condition than in the non-hydrated condition at the end of exercise (*P* <0.05), whereas Japanese participants showed no difference between the two hydration conditions. Hydration induced a greater total sweat loss in both groups (*P* <0.05), and the percentage of body weight loss in hydrated Malaysians was significantly less than in hydrated Japanese (*P* <0.05). A significant interaction between groups and hydration conditions was observed for the percentage of mean cutaneous vascular conductance during exercise relative to baseline (*P* <0.05).

**Conclusions:**

The smaller reduction in plasma volume and percentage body weight loss in hydrated Malaysians indicated an advantage in body fluid regulation. This may enable Malaysians to reserve more blood for circulation and heat dissipation and thereby maintain lower rectal temperatures in a hydrated condition.

## Background

During long-duration heat exposure, continuous sweating can induce hypovolemia and hyperosmolality because of the hypotonic nature of sweat. It has been reported that both hypovolemia and hyperosmolality inhibit thermoregulatory responses such as sweating and cutaneous vasodilation [ 1- 3]. Many studies have observed a greater rise in core body temperature during exercise in the heat when participants are hypohydrated compared with euhydrated [ 2- 5]. During exercise over a long duration, particularly in hot environments, either hypovolemia or blood distribution to the skin and working muscles can reduce central blood volume [ 6], which may elicit a decrease in stroke volume and an increase in heart rate compared to a euhydrated condition [ 1, 2, 4, 5, 7, 8]. On the other hand, Fritzche et al. [ 9] suggested the reduction in stroke volume was due to an increase in heart rate, and not cutaneous circulation.

The International Union of Physiological Sciences Thermal Commission glossary [ 10] defined the terms “acclimation” and “acclimatization” based on the assigned meanings of Hart [ 11] and Eagan [ 12]; they used the term acclimation to describe the adaptive changes that occur within an organism in response to experimentally induced changes in particular climatic factors, and the term acclimatization to describe the adaptive changes that occur within an organism in response to changes in the natural climate. In this study, we defined “short-term heat acclimation” as adaptive changes to experimentally induced, repeated exposure to heat or exercise training, and “long-term heat acclimatization” was defined as adaptive changes to the natural climate, especially in the case of indigenes living in a hot climatic region. It is well known that short-term, repeated exposure to heat or exercise training increases resting plasma volume [ 13- 17] and suppresses an elevation in the heart rate during exercise [ 14, 15, 18]. People acclimated to heat in the short-term have a smaller reduction in plasma volume reduction for a given body water deficit than those who are unacclimated [ 19]. However, it is difficult to apply the findings observed after short-term heat acclimation to the responses of long-term heat acclimatized populations since the general alteration in sweat response is different in short- and long-term heat adaptation [ 20, 21]. After short-term heat acclimation, a higher sweat rate is commonly observed [ 14, 18, 22, 23], whereas a number of studies on long-term heat acclimatization have reported a lower sweat rate among tropical natives compared with temperate natives during heat stress [ 20, 24- 26]. This reduced sweat response is considered to facilitate the efficiency of plasma preservation, which enables better heat tolerance for body fluid regulation than is the case for temperate natives [ 20, 24, 27]. However, no previous study has clearly compared body fluid regulation or osmoregulation between tropical and temperate indigenes [ 21].

Recently, our research group reported a significantly smaller increase in rectal temperature (*T*_re_) during passive heat exposure and exercise in the heat, in Malaysians compared with Japanese [ 28- 31]. A greater increase in the hand skin temperature in Malaysian participants partly explained the smaller increase in *T*_re_ since this acral body part is more vulnerable to heat loss due to its higher surface area to mass ratio and large number of arteriovenous anastomoses [ 32]. Further, a greater core-skin temperature gradient in Malaysians might facilitate heat transfer from the core to skin through blood circulation, which is a product of blood flow, the core-skin temperature gradient, and the specific heat of blood [ 33]. These heat dissipation responses indicated the possibility that tropical indigenes have an advantage in blood distribution and central blood volume control during prolonged exercise in the heat [ 29]. However, no previous studies have definitively established that blood volume and cardiovascular response offer tropical natives an advantage in body fluid regulation. Furthermore, no study to date has compared the effect of hypohydration on body fluid regulation or thermoregulation of tropical and temperate natives during heat exposure by testing hypohydrated and euhydrated conditions.

A recent study reported that fluid ingestion was more effective at preventing hyperthermia in trained vs. untrained individuals exercising in the heat [ 34]. It is not clear whether a similar advantage of hydration can be seen in long-term heat acclimatized populations. Comparative analyses between a non-hydrated condition, which was partly reported [ 29], and a hydrated condition, may clarify an advantage in body fluid regulation among long-term heat-acclimatized populations. The dehydration level in the non-hydrated condition was relatively mild (less than 2% of body weight; 1.77% and 1.61% in Japanese and Malaysians, respectively) [ 29] compared to previous studies testing the effect of hypohydration (3–5% of body weight) on thermoregulatory responses [ 1- 3]. However, when testing the effect of short-term heat acclimation, core temperature rose similarly in acclimated and non-acclimated individuals when they were hypohydrated at 5% of their body weight [ 3]. On the other hand, a less rapid rise in core temperature in the heat-acclimated subjects was observed when they exercised while euhydrated [ 3] or in a state of 1.5–2.0% mild hypohydration [ 35]. Additionally, Ikegawa et al. [ 36] reported that the enhanced cutaneous vasodilation, which occurs after short-term aerobic training, was blunted when participants were dehydrated (3% of body weight). Thus, the extent to which dehydration demonstrates an advantage in body temperature and fluid regulation in heat-acclimated and -acclimatized individuals may have a limit. Since acclimation/acclimatization in thermoregulatory response appears to be specific and confined to the environmental stress and physiological responses during the acclimation/acclimatization process [ 37], the advantage in thermoregulatory response of thermally acclimatized populations may be masked when the test condition is too severe compared with the acclimatized level. Thus, it is reasonable to test tropical indigenes for an advantage in body fluid regulation at the range of dehydration which they are accustomed.

The present study investigated the effect of hydration differences on body fluid regulation and heat dissipation responses during exercise in a hot and humid environment between tropical native Malaysians and temperate native Japanese. It was hypothesized that Malaysian participants have an advantage in body fluid regulation as a consequence of a smaller reduction in plasma volume compared to Japanese participants, and when hydrated, this may enable Malaysians to distribute more blood to the skin for heat dissipation.

## Methods

### Participants

Ten Malaysian males (tropical natives) from Kota Bharu, Malaysia, and 10 Japanese males (temperate natives) from Fukuoka, Japan, participated in this study. Kota Bharu in Malaysia (6º8′N, 102º15′E) is located in a tropical zone with dry and wet seasons, minimal seasonal variation, and an annual ambient temperature (*T*_a_) of 26°C with a 80–90% relative humidity (RH). Fukuoka in Japan (33º35′N, 130º24′E) is located in a temperate zone with hot summers and cold winters with an annual *T*_a_ of 16°C and 65–80% RH. During the three months prior to the experiment, the *T*_a_ and RH in Kota Bharu were 25°C and 84%, respectively, and in Fukuoka they were 7°C and 67%, respectively. All Japanese participants in this study had an air-conditioning system in their home, while no Malaysian participants did. Thus, the two groups were differently acclimatized to heat in their daily life. Additionally, differences in exposure to cold during their acclimatization process may influence thermoregulatory responses to heat. Most of the participants were recent or current university students who undertook regular exercise. The two groups in this study were matched in height, body weight, body surface area (SA), and peak oxygen consumption $\left(\dot{V}O_{2\text{peak}}\right)$ to avoid confounding factors related to differences in morphological characteristics and endurance training adaptation. Physical characteristics, reported as the mean (± standard error [SE]), for Japanese vs. Malaysian participants included age: 20.7 ± 0.3 vs. 22.3 ± 0.5 years; height: 168.9 ± 1.4 vs. 167.9 ± 1.7 cm; body weight: 63.0 ± 1.5 vs. 65.3 ± 3.6 kg; SA: 1.72 ± 0.03 vs. 1.74 ± 0.05 m^2^; SA/body weight: 0.027 ± 0.000 vs. 0.027 ± 0.001 m^2^ · kg^-1^; and $\dot{V}O_{2\text{peak}}:$ 41.8 ± 1.5 vs. 39.6 ± 1 mL · min^-1^ · kg^-1^. SA was estimated using the equation by DuBois and DuBois [ 38]. Malaysian participants were invited to stay in Japan for 10 days in early spring to participate in this study. Since the time difference between the countries is one hour, the jet-lag effect was considered negligible. All experimental protocols in this study were approved by the Institutional Review Board of Kyushu University. All participants were informed of the experimental procedures and gave their written informed consent before participation.

### Procedures

Prior to the main experiment, participants performed an incremental cycling exercise using an electrically-braked, semi-recumbent cycle ergometer (Aerobike 2100R, Combi wellness, Japan) to determine $\dot{V}O_{2\text{peak}}$ in a climatic chamber at *T*_a_ of 25°C with 50% RH. Each participant was required to pedal at 60 rpm starting from 40 W, and to gradually increase by 20 W every 2 min until volitional exhaustion. Expired gas was continuously measured by an automated respirometer (AE-300S, Minato Medical Science, Japan). $\dot{V}O_{2\text{peak}}$ was calculated by averaging data over the 30 sec prior to the participant becoming exhausted. The $\dot{V}O_{2\text{peak}}$ test was undertaken by Malaysian participants on the second or third day after their arrival in Fukuoka.

Two trials of the main experiment were conducted in the same climatic chamber at least three days after the $\dot{V}O_{2\text{peak}}$ test. In the non-hydrated trial, participants drank no water during the experiment. In the hydrated trial, participants drank 3 mL of water per kg body weight [ 39] at four intervals (1 min before exercise, and 15, 35, and 55 min after exercise began). The water temperature was maintained at 37°C, which likely had a negligible effect on body temperature, but no physiological measurement was conducted to test this assumption. The total volume of water intake (per kg body weight) was 755 ± 19 mL (12 mL · kg^-1^) and 783 ± 43 mL (12 mL · kg^-1^) for Japanese and Malaysian participants, respectively. These trials were performed in random order, and were conducted at the same time of day to reduce circadian effects. Half of the participants in each group performed their experiments before noon and the other half did so in the afternoon. Participants wore only shorts during the experiment. After their body weight was measured, participants entered a chamber which was controlled at 28°C *T*_a_ and 50% RH, and rested in a semi-recumbent position for at least 40 min for stabilization. During this period, a Teflon venous catheter, fitted with a three-way stopcock for blood sampling, was inserted into a forearm vein and all other instruments (described later) were attached to the participants. Thereafter, all measurements were started from the 10-min baseline. Following this, the participants started a semi-recumbent cycle exercise at 60 rpm for 60 min at 55% maximal exercise intensity based on their pre-determined $\dot{V}O_{2\text{peak}}$. There was no difference in absolute work load between groups (104.9 ± 12.6 W and 100.9 ± 12.1 W for Japanese and Malaysian participants, respectively; *P* = 0.48). Mean oxygen uptake $\left(\dot{V}O_{2}\right)$ between the 50 to 55-min period during the exercise phase was not different between both groups and conditions (20.7 ± 0.8 and 21.7 ± 0.8 mL · min^-1^ · kg^-1^ in hydrated and non-hydrated Japanese; 21.2 ± 1.0 and 19.9 ± 0.5 mL · min^-1^ · kg^-1^ in hydrated and non-hydrated Malaysians). After the 10-min period to establish a baseline measurement while in the thermoneutral climatic chamber (28°C *T*_a_ and 50% RH), participants started pedaling while there was a gradual increase in *T*_a_ and RH to 32°C and 70% RH, respectively, during the first 10 min of exercise. After the 60 min of exercise, participants passively recovered for 30 min in a semi-recumbent position. Following the protocol, participants’ body weight was measured before and after urination. The night before, and two hours prior to the experiment, each participant was given 500 mL and 300 mL of drinking water, respectively, to increase the probability that they would begin their protocols in a euhydrated state as shown in a previous study [ 15]. Additionally, urine specific gravity before starting the experiment was measured for the assessment of hydration status [ 40]. The specific gravity of urine was in the euhydrated range (below 1.030), reported by Armstrong [ 40], and no difference between both groups and conditions was evident (1.021 ± 0.002 and 1.017 ± 0.003 in hydrated and non-hydrated Japanese; 1.017 ± 0.003 and 1.014 ± 0.004 in hydrated and non-hydrated Malaysians). Participants were required to abstain from strenuous exercise for at least 48 hours prior to each test. No caffeinated drinks, alcoholic beverages, or drugs were allowed from the night before the experiment, and participants were prohibited from taking food for at least 2 hours prior to the experiment.

### Measurements

Rectal temperature (*T*_re_) was measured by a thermistor probe inserted 13 cm beyond the anal sphincter. Skin temperature was measured by thermistor probes at ten body sites (forehead, chest, abdomen, back, upper arm, forearm, hand, thigh, calf, and foot). The *T*_re_ and skin temperatures were monitored every two seconds by a data logger (LT-8A, Gram Corporation, Japan), and averaged every minute for subsequent data analyses. Mean skin temperature $\left({\bar{T}}_{\mathrm{sk}}\right)$ was estimated from a modified Hardy and DuBois’ equation [ 41], as follows:

$$\begin{array}{l} {\bar{T}}_{\mathrm{sk}}=0.07T_{\text{forehead}}\\ \;+0.35\left(T_{\text{chest}}+T_{\text{abdomen}}+T_{\text{back}}\right)/3\\ \;+0.14\left(T_{\text{upper}\;\text{arm}}+T_{\text{forearm}}\right)/2+0.05T_{\text{hand}}\\ \;+0.19T_{\text{thigh}}+0.13T_{\text{calf}}+0.07T_{\text{foot}}. \end{array}$$

Skin blood flow (SkBF) in the back (left scapula) and left forearm (ventral, mid-anterior) were measured by laser Doppler flowmetry (FLO-C1, OMEGAWAVE, Japan). The SkBF data were sampled using an A/D converter (Powerlab/16SP, AD Instruments, Australia) and were recorded at one-second intervals using a personal computer (PC-AL70G, SHARP, Japan). To minimize the artifact due to movement of the laser Doppler probe, participants were familiarized with the protocol of cycling while keeping their upper body and arms as stable as possible, with their upper body lying on the seat of a semi-recumbent ergometer and their arms placed on fixed tables. Additionally, in the subsequent analysis, the artifact observed in the raw data was eliminated using a 10-second median filter.

Arterial blood pressure was measured at the upper arm using an automated sphygmomanometer (STBP-780B, COLIN, Japan); the right arm was placed on a table at each participant’s heart level. Blood pressure measurement was repeated twice every 10 min, and the mean value was used in the following analysis. Mean arterial blood pressure (MAP) was calculated from systolic blood pressure (SBP) and diastolic blood pressure (DBP) (MAP = DBP + (SBP – DBP)/3). Cutaneous vascular conductance (CVC) in the back and forearm was calculated as SkBF at each site divided by MAP. Additionally, SkBF in the back and forearm were averaged and the mean CVC of the two sites were calculated. Since the laser Doppler flowmetry signal does not provide an absolute measurement of blood flow, all the CVC values during exercise and recovery were normalized relative to baseline levels which were measured during rest at 28°C *T*_a_ and designated as 100%. Participants entered the same chamber and rested in a semi-recumbent position for at least 40 min before baseline measurements started. The result was expressed as the percentage of baseline CVC (%CVC). Heart rate was measured continuously throughout the experiment using a heart rate monitor (RS400, Polar Electro Oy, Finland), and averaged for every minute.

Participants’ body weight was measured before the experiment, immediately after the recovery period, and after urination using a calibrated scale (ID2, Mettler-Toledo, Germany) with a minimum calibration of 1 g. The percentage of body weight loss was calculated from body weight before the experiment and after urination, excluding insensible body-weight loss during the pre-experiment preparation. Total sweat loss was estimated from the body weight difference prior to the experiment and immediately after, subtracting insensible body-weight loss, water intake, and the blood sampling volume. A further experiment was used to measure insensible body-weight loss. This was estimated using the difference in body weight measured before and after a two hour rest in a room maintained at a *T*_a_ of 28°C and 50% RH. We then calculated the total sweat loss caused by the exercise-induced heat stress. Urine weight during the experiment was calculated from the reduction of body weight after urination. The specific gravity of urine was measured pre- and post-experiment by a refractometer (PAL-09S, ATAGO Co. Ltd., Japan).

Blood samples (10 mL each) were collected from a left side forearm vein through a Teflon venous catheter at baseline, 5 min and 60 min after the start of exercise, and at the end of recovery. The stabilization period (>40 min) prior to the first sampling was long enough to complete equilibrium of plasma volume change with movement from a standing to recumbent position [ 42]. Concentrations of plasma sodium (Na^+^), hemoglobin (Hb), and hematocrit (Hct) were analyzed. Na^+^ was determined by the ion selective electrode (AU5400, Beckman Coulter, Inc., USA). Hb was determined by the SLS hemoglobin method. The percentage change in plasma volume (∆PV) was estimated from the changes in Hct and Hb at baseline and during exercise according to the Dill and Costill equation [ 43] [∆PV = 100 (Hb_base_/Hb_exer_) (1–Hct_exer_/100)/(1–Hct_base_/100) –100]. Blood samples could not be obtained from two Malaysian participants due to their discomfort. Participants were asked to categorize their thirst sensation (0 = not at all, 1 = slightly thirsty, 2 = thirsty, 3 = very thirsty) at the same time when the blood samples were collected.

### Statistics

Continuously measured data (*T*_re_, ${\bar{T}}_{\mathrm{sk}}$, and %CVC) were analyzed by mixed factorial two-way (participant groups × hydration condition) analysis of variance (ANOVA), followed by a post-hoc test, using a representative value for each baseline, exercise, and recovery period. Temperature at the end of each phase was set as a representative value for *T*_re_ and ${\bar{T}}_{\mathrm{sk}}$. The mean data collected over 30 to 60 min and 65 to 90 min was used as the representative value for each exercise and recovery phase for %CVC and heart rate. A mixed factorial two-way ANOVA (groups × hydration condition) was conducted for the following variables: the change of Na^+^ relative to the baseline (∆Na^+^) and ∆PV at each measurement point; percentage of body weight loss, total sweat loss, urine weight, and specific gravity of urine. Following the identification of a main effect, a paired Student’s *t*-test was used to examine differences between the two hydration conditions within each group, or unpaired Student’s *t*-tests were used to determine the difference between groups for each hydration condition. Additionally, to analyze the time course of ∆Na^+^ and ∆PV (at baseline, 5 and 60 min after commencement of exercise, and 30-min recovery) a repeated measures ANOVA followed by a pair-wise Tukey’s post-hoc test was conducted. Significant differences were established at *P* <0.05. All data were presented as mean values and the SE.

## Results

### Body temperature

**Figure 1:**
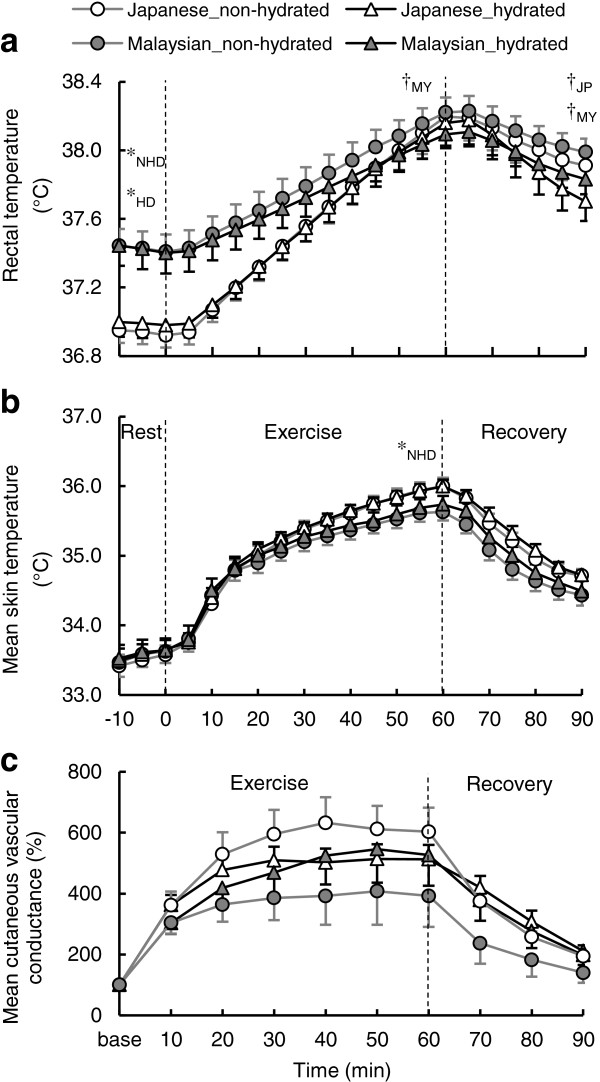
**Time course of rectal temperature (a), mean skin temperature (b), and mean cutaneous vascular conductance (c) during the experiment.** Values are the mean ± SE for 10 Japanese and 10 Malaysians in both the non-hydrated and hydrated condition. Significant differences in each phase are shown between Japanese and Malaysians in non-hydrated (*_NHD_) or hydrated conditions (*_HD_), and between non-hydrated and hydrated conditions in Japanese (†_JP_) or Malaysian groups (†_MY_).

The *T*_re_ in Malaysians was significantly higher at baseline compared with Japanese in both hydrated and non-hydrated conditions (*P* <0.05), as shown in Figure 1a, but no difference was observed between hydrated and non-hydrated conditions in Japanese and Malaysians. After the 60 min of exercise, no group difference was observed in *T*_re_ in hydrated and non-hydrated conditions, but the Malaysian group showed a significantly lower *T*_re_ in the hydrated vs. the non-hydrated condition (*P* <0.05). This was due to a significantly smaller *∆T*_re_ in the hydrated compared with the non-hydrated condition in Malaysians (0.69 ± 0.07°C and 0.81 ± 0.05°C, respectively; *P* <0.05). After the 30 min recovery period, no group difference was observed in hydrated and non-hydrated conditions, but both the Malaysian and Japanese groups showed significantly lower *T*_re_ in the hydrated vs. non-hydrated condition (*P* <0.05). No difference was observed in ${\bar{T}}_{\mathrm{sk}}$ in hydrated or non-hydrated conditions at the baseline within each group, or between Malaysians and Japanese (Figure 1b). A significantly higher ${\bar{T}}_{\mathrm{sk}}$ in Japanese vs. Malaysians was observed at the end of the exercise for the non-hydrated condition (*P* <0.05). Only Malaysians showed a higher tendency in ${\bar{T}}_{\mathrm{sk}}$ at the end of exercise when in the hydrated vs. non-hydrated condition (*P* = 0.09). After that, ${\bar{T}}_{\mathrm{sk}}$ gradually decreased similarly in both groups and was not significantly different after the 30-min recovery.

### Fluid loss, hematologic status, and thirst sensation

The percentage of body weight loss (which included the ingested water) was significantly less in the hydrated condition in both groups (*P* <0.05) as shown in Table 1 in conjunction with other body fluid data. The percentage of body weight loss was significantly less in Malaysians than in Japanese in the hydrated condition (*P* <0.05), while no group difference was observed for the non-hydrated condition. Total sweat loss per kg body weight was significantly more in the hydrated vs. the non-hydrated condition for both groups (*P* <0.05, and sweat loss tended to be less in hydrated Malaysians than in hydrated Japanese (*P* = 0.08). Urine weight relative to body weight was not different between groups in hydrated and non-hydrated conditions, but urine weight was higher in Japanese when they were hydrated (*P* <0.05). Malaysians showed a trend towards a lower post experiment urine specific gravity than Japanese in the non-hydrated condition (*P* = 0.06). Only Japanese participants had a significantly lower urine specific gravity post experiment when in hydrated vs. non-hydrated conditions (*P* <0.05). The pre- to post-experiment change in urine specific gravity was not different between groups, but a significant difference was observed between hydration conditions in both groups (*P* <0.05).

**Table 1:** Body fluid loss during experiment

	**Body weight loss, %**		**Total sweat loss, g · kg**^**-1**^		**Urine weight, g · kg**^**-1**^		**Urine specific gravity**		**ΔUrine specific gravity**
Japanese non-hydrated	-1.77	(0.11)	15.3	(1.1)	1.8	(0.4)	1.022	(0.001)	0.005 (0.002)
Japanese hydrated	-1.04	(0.14) †	17.2	(1.3) †	4.6	(1.1) †	1.012	(0.002) †	-0.009 (0.004)†
Malaysian non-hydrated	-1.61	(0.09)	13.6	(0.8)	2.1	(0.4)	1.016	(0.002)	0.002 (0.002)
Malaysian hydrated	-0.66	(0.08) *†	14.4	(0.8) †	3.8	(0.7)	1.011	(0.003)	-0.006 (0.003)†

Values are the mean (SE) for 10 Japanese and 10 Malaysians in the non-hydrated and hydrated conditions. ΔUrine specific gravity is the change from pre- to post-experiment. *Significant difference between Japanese and Malaysian participants in the hydrated or non-hydrated condition. †Significant difference between the hydrated and non-hydrated condition in Japanese or Malaysians.

Plasma and thirst data are shown in Table 2. No difference was observed in ∆PV between groups throughout the experiment. Malaysians showed a significantly smaller reduction of plasma volume 60 min after starting exercise in the hydrated vs. non-hydrated condition (*P* <0.05). A similar rapid reduction of plasma volume was observed for both hydration conditions at 5 min after the onset of exercise (*P* <0.05); only non-hydrated Japanese showed a further significant reduction after 60 min of exercise (*P* <0.05). Only Malaysians in the hydrated condition recovered to a plasma volume comparable to their baseline plasma volume; while the other hydration condition resulted in a significant reduction in plasma volume at the end of the recovery period compared with baseline values (*P* <0.05). No difference was observed in ∆Na^+^ between groups throughout the experiment. Only Malaysians showed a significantly smaller ∆Na^+^ after 60 min of exercise in the hydrated vs. non-hydrated condition (*P* <0.05). Hydration reduced ∆Na^+^ in both Malaysian and Japanese participants at the end of the 30 min recovery period (*P* <0.05). Both Malaysians and Japanese showed a significantly lower thirst sensation in the hydrated vs. the non-hydrated condition after 60 min of exercise and after the 30 min recovery period (*P* <0.05). Malaysians showed a significantly lower thirst sensation at the end of exercise compared to Japanese participants in the non-hydrated condition (*P* <0.05).

**Table 2:** Change in plasma volume, plasma sodium concentration, and thirst sensation

	**Baseline**		**Exercise 5 min**		**Exercise 60 min**		**Recovery 30 min**	
∆Plasma volume, %								
Japanese non-hydrated			-6.1	(0.8) ‡	-9.6	(1.2) ‡#	-4.1	(0.8) ‡
Japanese hydrated			-5.6	(0.9) ‡	-8.0	(1.0) ‡	-4.5	(0.8) ‡
Malaysian non-hydrated			-5.2	(0.6) ‡	-7.8	(1.7) ‡	-3.6	(1.4) ‡
Malaysian hydrated			-5.3	(0.5) ‡	-5.6	(1.7) †‡	-1.0	(1.5)
∆Sodium, mmol · L ^-1^								
Japanese non-hydrated			1.5	(0.4) ‡	1.9	(0.3) ‡	1.4	(0.4) ‡
Japanese hydrated			1.8	(0.3) ‡	1.2	(0.4)	-0.1	(0.4) †
Malaysian non-hydrated			1.6	(0.4) ‡	2.3	(0.3) ‡	1.6	(0.5) ‡
Malaysian hydrated			1.3	(0.3) ‡	0.4	(0.2) †	-1.3	(0.6) †‡
Thirst sensation								
Japanese non-hydrated	0.2	(0.1)	0.8	(0.2)	2.2	(0.1)	1.7	(0.2)
Japanese hydrated	0.2	(0.1)	0.6	(0.1)	0.7	(0.3) †	0.5	(0.2) †
Malaysian non-hydrated	0.0	(0.0)	0.5	(0.1)	1.4	(0.2) *	1.3	(0.2)
Malaysian hydrated	0.2	(0.1)	0.4	(0.2)	0.1	(0.1) †	0.1	(0.1) †

Values are the mean (SE). The percentage change in plasma volume and plasma sodium concentration was analyzed in 10 Japanese and 8 Malaysians. *Significant differences between Japanese and Malaysians in the non-hydrated condition. †Significant difference between the hydrated and non-hydrated condition in Japanese or Malaysians at the same time points. ‡Significantly different from baseline within a trial. #Significant difference between 5 min and 60 min after starting exercise.

### Cardiovascular function

**Figure 2:**
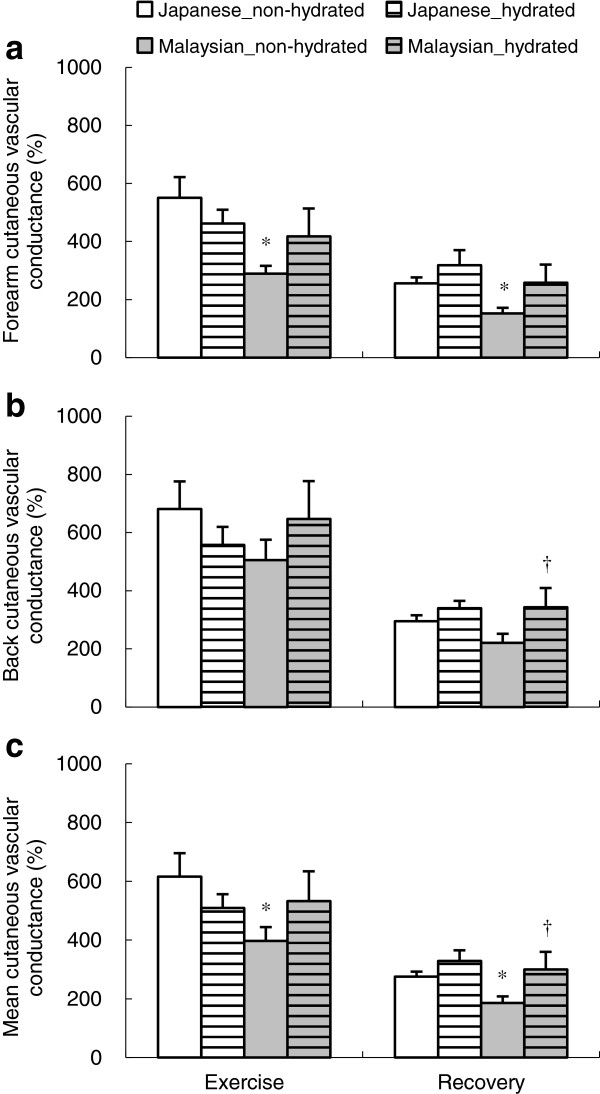
**The percentage of cutaneous vascular conductance relative to baseline during exercise and recovery phase in the forearm (a), the back (b), and the mean of the back and forearm (c).** Data means collected over 30 to 60 min and 70 to 90 min were used as the representative value for each exercise and recovery phase. Values are the mean ± SE for 10 Japanese and 10 Malaysians in both the non-hydrated and hydrated conditions. Significant differences in each phase are shown between Japanese and Malaysians in the non-hydrated condition (*), and between non-hydrated and hydrated conditions in the Malaysian group (†).

The 30- to 60-min averaged %CVC in the forearm during exercise was significantly higher in Japanese compared with Malaysians in the non-hydrated condition (*P* <0.05) (Figure 2a), but no group difference was observed in the hydrated condition. No group difference was observed in %CVC in the back for both hydrated and non-hydrated conditions (Figure 2b). Both groups showed no difference in the 30- to 60-min averaged %CVC in the back and forearm between hydrated and non-hydrated conditions. Mean %CVC of the back and forearm demonstrated a significant interaction of group and hydration conditions (*P* <0.05), as shown in Figure 2c. During the recovery period, the 70- to 90-min averaged %CVC in the forearm was significantly higher in the Japanese compared with the Malaysians in the non-hydrated condition (*P* <0.05), but no group difference was observed in the hydrated condition. %CVC in the back in both hydrated and non-hydrated participants was not different between groups. Only Malaysians showed a significantly higher %CVC in the back in the hydrated vs. non-hydrated condition (*P* <0.05) and a tendency toward a higher %CVC in the forearm in the hydrated condition during recovery (*P* = 0.09). Mean %CVC of back and forearm in the hydrated condition was significantly higher than the non-hydrated condition in Malaysians (*P* <0.05). Additionally, the time course of mean %CVC of the back and forearm is shown in Figure 1c.

**Figure 3:**
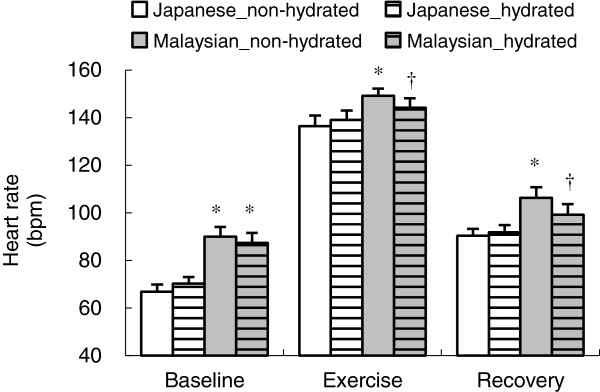
**Heart rate during baseline, and the exercise and recovery phase.** Data means collected over 30 to 60 min and 65 to 90 min were used as the representative value for each exercise and recovery phase. Values are the mean ± SE for 10 Japanese and 10 Malaysians in both the non-hydrated and hydrated conditions. Significant differences in each phase are shown between Japanese and Malaysians in the non-hydrated condition (*), and between non-hydrated and hydrated conditions in the Malaysian group (†).

The 30- to 60-min averaged heart rate during exercise and the 65- to 90-min averaged heart rate during the recovery period was significantly higher in Malaysians compared to Japanese participants in the non-hydrated condition (*P* <0.05) (Figure 3), but no group difference was observed in the hydrated condition. Only Malaysians showed a significantly lower heart rate when in the hydrated vs. non-hydrated condition, during both exercise (*P* <0.05) and recovery periods (*P* <0.05).

## Discussion

This study examined the effects of water intake on body fluid and temperature regulation in Malaysian and Japanese males exercising in humid heat. The major findings were that hydration induced a significantly smaller decrease in the plasma volume during exercise in the Malaysian vs. the Japanese participants. This may enable Malaysians to reserve more circulating blood for heat dissipation and to maintain a smaller rise in *T*_re_ in hydrated conditions.

During the 60-min exercise session at 55% $\dot{V}O_{2\text{peak}}$ intensity in a hot and humid environment, the same amount of hydration per kg body weight suppressed a rise in *T*_re_ compared to the non-hydrated condition in Malaysian but not in Japanese participants (Figure 1a). This indicates that the Malaysian participants better utilized the limited amount of water intake for a heat dissipation response. It has been reported that short-term heat acclimation lowers core body temperature change during exercise in a euhydrated [ 3] or 1.5–2.0% mild hypohydration condition [ 35]; however, no acclimation effect was observed for severe hypohydration (5% of body weight [ 3]). There may be a limit of dehydration to show an advantage in body temperature and fluid regulation in heat acclimated and acclimatized individuals. Daily euhydration variability has been reported in heat and exercise conditions (±0.48% of body weight [ 44]). A mild level of dehydration was noted in the present study (less than 2% of body weight) and seems to be in a reasonable range to test the effect of long-term heat acclimatization on the body fluid regulation. In this study, Malaysian participants showed significantly higher *T*_re_ at rest. This observation differs from the well-known characteristics of short-term heat acclimation showing lower resting core temperature [ 15, 36]. However, in the case of long-term heat acclimatized populations, several studies support our observation showing higher resting core body temperatures in tropical indigenes [ 31, 45- 47]. There might be some different mechanism setting their resting core temperature after short-term acclimation or long-term heat acclimatization.

In this study, a similar rapid reduction of plasma volume was observed in all conditions at 5 min after the onset of exercise (Table 2). This was due to the shift of body fluid from the intra- to extra-vascular space for contracting muscles before any reduction of body fluid content [ 48, 49]. After the 60-min exercise session, only the Malaysians in the hydrated condition showed a significantly smaller reduction in plasma volume (*P* <0.05). This may enable Malaysians to reserve more circulating blood to distribute for heat dissipation as shown in the significant interaction of groups and hydration conditions in percentage of mean cutaneous vascular conductance during exercise (Figure 2c). In Malaysians, a significantly lower heart rate when in the hydrated condition was partly explained by the smaller reduction in plasma volume (Figure 3). The greater increase in heart rate in the non-hydrated condition may be due to a stroke volume reduction induced by a decrease in plasma volume [ 1, 2, 4, 7, 8], or the higher core body temperature may elicit a greater increase in heart rate [ 50] resulting in a reduction of stroke volume [ 9]. On the other hand, Japanese participants showed no difference in their plasma volume (Table 2) or core body temperature changes (Figure 1a) in hydrated and non-hydrated conditions. Recently, Mora-Rodriguez et al. [ 34] reported that fluid ingestion is more effective in preventing hyperthermia in trained vs. untrained individuals. In their study, at 40% $\dot{V}O_{2\text{peak}}$, fluid ingestion reduced *T*_re_ in both trained and untrained individuals, whereas, at 60% $\dot{V}O_{2\text{peak}}$, this only occurred in the trained group. The reduction of plasma volume was also ameliorated in both groups when they exercised at 40% $\dot{V}O_{2\text{peak}}$, whereas at 60% $\dot{V}O_{2\text{peak}}$, only the trained group was similarly affected. Similar results in *T*_re_ and plasma volume in Malaysian and Japanese groups were observed in the present study which tested participants at 55% $\dot{V}O_{2\text{peak}}$. Thus, the heat strain (based on exercise intensity and environmental conditions) of the present study was in a range that showed that heat-acclimatized Malaysians derive more physiological benefit from hydration vs. Japanese, but the heat strain might be excessive for non-acclimatized Japanese.

The smaller reduction in plasma volume observed in hydrated Malaysians can be partly explained by a lower percentage of body weight loss when in the hydrated condition (Table 1). The percentage of body weight loss was significantly less in Malaysians compared with Japanese in the hydrated condition. This indicates the body fluid loss in hydrated Japanese is higher compared with hydrated Malaysians due to the combined effects of the total sweat loss being slightly more, and urine weight observed in hydrated Japanese being slightly increased (Table 1). The greater sweat loss in the hydrated condition was consistent with previous studies [ 3]. Hydration increased total sweat loss by 1.8 ± 0.6 g · kg^-1^ in Japanese vs. 0.8 ± 0.3 g · kg^-1^ in Malaysians. However, since the sweat in the non-hydrated condition could be assumed to be sufficient to wet the overall skin surface [ 29], the increase in total sweat loss in the hydrated condition did not induce further evaporative heat loss. Additionally, an increase in urine volume reduces circulating blood volume. Thus, the greater body fluid loss observed in the hydrated Japanese seemed to be a wasteful overproduction of sweat and urine. In contrast, the Malaysians demonstrated more efficient fluid regulation and this maintained plasma volume; the smaller rise in sodium concentration occurred as excessive fluid loss was prevented despite a moderate dehydration occurring in the hydrated condition. The Malaysian participants may have had more body fluid at baseline considering it has been shown that a higher initial body water percentage has been reported in long-term heat-acclimatized Bantu indigenes relative to Europeans [ 51]. Additionally, Malaysian participants demonstrated less thirst sensations (Table 2) which might indicate that they have acclimatized to maintain their body fluid by restricting urine excretion rather than by increasing voluntary water intake, which has been observed after short-term heat acclimation [ 44]. Moreover, a lower electrolyte concentration in the sweat of tropical natives has been previously reported [ 20, 24, 52]. A greater solute retention in the plasma in heat-acclimated individuals could cause more fluid to move from the intra- to the extracellular compartment, depending on the osmotic gradient between these spaces [ 19].

The advantage in body fluid regulation by maintaining plasma volume, which we observed in hydrated Malaysians, might enable them to reserve more circulating blood for heat dissipation. It has been suggested that an expanded plasma volume causes an increased cutaneous blood flow [ 1, 2, 4]. Additionally, it has been reported that hyperosmolality inhibits thermoregulatory responses such as sweating and cutaneous vasodilation [ 1- 3]. The smaller increase in the plasma sodium concentration observed in the hydrated Malaysians might ease the suppression of thermoregulatory responses. The significant interaction of groups and hydration conditions in the percentage of mean cutaneous vascular conductance during exercise (*P* <0.05), as shown in Figure 2c, may indicate an advantage gained by blood distribution to the skin when the Malaysian participants are hydrated. At the end of exercise, mean skin temperature tended to be higher in hydrated Malaysians (*P* = 0.09), whereas the Japanese did not show any difference between the hydration conditions (Figure 1b). The heat dissipation response, attributed to an advantage in body fluid regulation in hydrated Malaysians, may induce a lower *T*_re_ than in a non-hydrated condition. The repeated exposure to a climate with humid heat might be the reason Malaysians can adapt to more economically manage their body temperature and fluid regulation by maintaining plasma volume to reserve more circulating blood for heat dissipation. Since evaporative heat loss via sweating is relatively inefficient in humid heat compared with dry heat, the environmental conditions in this study might elicit an advantage in Malaysian participants’ thermoregulatory responses. It was reported that a sinusoidal wave indicating the normal daily water content (euhydration) is regulated to within ±0.22% of body weight in normal temperate conditions, whereas within ±0.48% in heat and exercise conditions [ 44]. Since the annual ambient temperature and humidity is higher in Malaysia (26°C with 80–90% RH) than in Japan (16°C with 65–80% RH), the authors speculate that more daily dehydration occurs in Malaysians vs. Japanese and may be the reason Malaysians exhibit an advantage in body fluid conservation.

One of the limitations in this study was a higher resting heart rate observed in Malaysian compared with Japanese participants. There was no clear reason for the higher heart rate in Malaysians; it might be due to the mental stress associated with visiting and staying in Japan. It would be possible that this higher heart rate causes more vasoconstriction in the skin. Thus, this may have affected the results in this study.

## Conclusions

In conclusion, a significantly smaller reduction in plasma volume during exercise and a smaller percentage of body weight loss in hydrated Malaysians compared with Japanese participants indicates an advantage in body fluid conservation in Malaysians. This may enable Malaysians to reserve more circulating blood for heat dissipation and to maintain a smaller increase in *T*_re_ when hydrated.

## Abbreviations

CVC: Cutaneous vascular conductance; %CVC: Percentage of baseline CVC; MAP: Mean arterial blood pressure; ∆PV: Percentage change in plasma volume; RH: Relative humidity; SA: Surface area; SkBF: Skin blood flow; *T*_a_: Ambient temperature; *T*_re_: Rectal temperature; ${\bar{T}}_{\mathrm{sk}}$: Mean skin temperature; $\dot{V}O_{2\text{peak}}$: Peak oxygen consumption

## Competing interests

The authors declare that they have no competing interests.

## Authors’ contributions

HW, YT and MS designed the study. HW, TW, JYL, NH and MS conducted the experimental work. HW analyzed data, prepared figure and drafted manuscript. All authors participated in data interpretation and revised the manuscript. The final version of manuscript was approved by all authors.
